# The Passive, The Active, and The Seeker: A Descriptive Taxonomy of Substance Use Disorder Patients Based on Digital Behaviors

**DOI:** 10.1192/j.eurpsy.2026.10906

**Published:** 2026-07-31

**Authors:** E. M. Bahri, J. Nasri, S. Hleli, M. Bennsir, W. Abdelghaffar

**Affiliations:** Hope Department for Addiction Care, Jbel Ouest Health Center, Zaghouan, Tunisia

## Abstract

**Introduction:**

Patients with substance use disorders are not a monolith in their digital behaviors. The digital ecosystem presents a dual reality for this population, acting as both a potential vector of risk—through exposure to triggering content and drug market access—and a vital channel for support and health information. Understanding this complex relationship is crucial for modern addiction care.

**Objectives:**

This study aims to move beyond simple prevalence to create a descriptive typology, classifying patients based on how they use their smartphones in relation to their substance use.

**Methods:**

This was a cross-sectional, descriptive, observational study conducted on a cohort of 60 outpatients with Substance Use Disorders, recruited from the Hope Department for Addiction Care at the Jbel Ouest Health Center. Each participant was invited to fill in a form composed of : A sociodemographic and clinical data collection form, a purpose-built questionnaire designed to explore digital behaviors related to smartphone and social media use, comprising nine closed and semi-open questions and the translated and validated Literary Arabic version of the Smartphone Addiction Scale – Short Version (SAS-SV).Data was collected and analyzed using IBM SPSS Statistics software, version 27.

**Results:**

The sample (N=60) was predominantly male (n=56, 93.3%) and single (n=46, 76.7%). The participants’ ages ranged from 18 to 52 years, with a mean age of 29.0 (SD = 8.72). Most participants had a secondary education level (n=44, 73.3%) and were day labourers (n=20, 33.3%) or unemployed (n=14, 23.3%). Over one-third of participants (n=23, 38.3%) met the criteria for smartphone addiction according to the SAS-SV scale.

The analysis of digital behaviors revealed a clear typology among patients. The largest group was the “Help Seekers” (40%, n=24), who primarily used their smartphones to search for treatment information and online support. A significant portion were “Passive Consumers” (15%, n=9), exposed to drug-related content but not engaging further. Smaller groups included “Active Actors” (5%, n=3), who used their phones for transactions and dedicated forums, and “Neutral” users (10%, n=6), who reported none of these behaviors. Data was missing for 30% (n=18) of the sample.

**Conclusions:**

This patient-centered typology reveals distinct patterns of digital behavior, suggesting different motivations and risks. Classifying patients into these archetypes can help clinicians personalize interventions, from providing targeted psychoeducation for Passive Consumers to offering safe technology alternatives for Help Seekers and more stringent monitoring for Active Actors.

**Disclosure of Interest:**

None Declared

